# High Speed Sintering of Polyamide 12: From Powder to Part Properties

**DOI:** 10.3390/polym16243605

**Published:** 2024-12-23

**Authors:** Jan Kemnitzer, Marco Wimmer, Anna Tarasova, Frank Döpper

**Affiliations:** 1 Fraunhofer Institute for Manufacturing Engineering and Automation IPA, Universitaetsstrasse 9, 95447 Bayreuth, Germany; marco.wimmer@ipa.fraunhofer.de (M.W.); frank.doepper@ipa.fraunhofer.de (F.D.); 2 Chair Manufacturing and Remanufacturing Technologies, University of Bayreuth, Universitaetsstrasse 30, 95447 Bayreuth, Germany

**Keywords:** additive manufacturing, powder bed fusion of polymer, high-speed sintering, laser sintering, multi jet fusion, polyamide 12

## Abstract

High Speed Sintering (HSS) is an additive manufacturing process with great potential to produce complex, high-quality polymer parts on an industrial scale. However, little information is currently available on the characteristics of the powder materials used and the part properties that can be achieved. This is also the case for the standard material polyamide 12 (PA 12) and the first commercially available HSS machine, the VX200 HSS. The aim of this study is, therefore, to provide the first comprehensive overview of the properties of PA 12 parts manufactured with the VX200 HSS and the characteristics of the PA 12 powder. This includes the analysis of the influence of part orientation and part position in the build job. To characterize the powder, particle size distribution, particle shape, thermal properties and powder flowability were analyzed. To characterize the parts, density and various thermal, mechanical and geometrical properties were analyzed. In summary, the powder material and part properties are largely similar to those of other Powder Bed Fusion of Polymer (PBF/P) processes. However, there are also significant differences for some part properties. It was also determined that the anisotropy of parts is very low compared to that of many other additive manufacturing processes.

## 1. Introduction

Additive manufacturing has developed significantly in recent years, offering a wide range of possibilities for the resource-efficient production of single and low-volume parts with highly complex geometries. Additive manufacturing processes in the category of Powder Bed Fusion of Polymer (PBF/P) have great potential for use as serial processes for the production of polymer parts on an industrial scale [1]. Currently, the most established and widely used PBF/P process in the industry is Laser Sintering (LS). However, the ’younger’ and much less researched PBF/P process, High Speed Sintering (HSS), has technical, environmental and economic advantages over LS [2,3].

### 1.1. Process of the HSS

**Figure 1:**
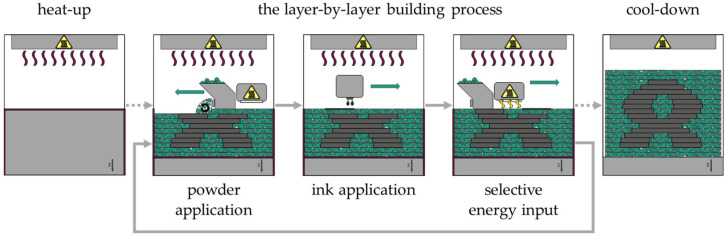
Schema of the HSS process.

In the HSS process, a polymer powder is selectively melted by a sintering lamp using a Radiation Absorbing Material (RAM), which is selectively applied to the surface of the powder bed. The HSS process is shown schematically in Figure 1. As with other PBF/P processes, the heat-up is essential for processing the polymer powder. This is because the polymer powder must be brought to a temperature just below its melting point to avoid thermal disturbances in the process. In the first step of the layer-by-layer building process, the recoater applies a thin layer of polymer powder to the surface of the powder bed (powder application). A print head then selectively applies ink to the areas to be fused (ink application). The ink contains carbon black, which acts as a RAM. The sintering lamp is then moved across the powder bed surface (selective energy input). The temperature of the inked powder is raised above its melting point due to the RAM’s high absorption capacity. Due to the lower absorption of the radiation from the sintering lamp, the polymer powder not wetted by the ink does not fuse and remains loose, acting as a support. These steps—powder application, ink respective RAM application and selective energy input—are repeated until all parts have been produced. Loose powder and parts must be cooled (cool-down) before parts can be removed from the powder bed [4,5,6].

The HSS process is similar to the Multi Jet Fusion (MJF) process, which is currently more widespread. The main differences between HSS, as implemented by voxeljet, and MJF, as implemented by Hewlett–Packard (HP), are as follows: In MJF, two inks (fusing agent, detailing agent) are applied to the powder bed. The function of the fusing agent is comparable to that of the ink used in HSS. In both cases, carbon black is used as the RAM. In contrast to HSS, where a petroleum-based ink is used, the fusing agent is water-based. The detailing agent is also a water-based ink that is selectively applied to the surface of the powder bed to control its temperature distribution. In addition, the sintering lamp and overhead heater used in each process have different wavelength ranges. In HSS, the sintering lamp operates in the near-infrared range, and the overhead heater operates in the mid-infrared range [7]. In MJF, both lamps operate in the near-infrared range.

### 1.2. Potential of the HSS

The planar energy input of HSS has several advantages over the punctual or line-by-line energy input of LS. As a result of the planar energy, a significantly longer duration of selective energy input per unit area can be achieved with high productivity. This, in turn, allows for the processing of polymers with high melt viscosity [3] and results in significantly lower maximum temperatures, which means that the degradation of many polymers can be reduced or avoided. In addition, this generally enables a higher recycling rate of the materials [3]. Furthermore, a constant deposition time is achieved regardless of the area of a layer to be selectively melted. This allows for high productivity and reproducibility as well as the economical scaling of the system technology [2,3]. Because of these advantages, HSS enables the cost-effective production of high-quality polymer parts on an industrial scale.

### 1.3. State of the Research on HSS

Most of the published studies on HSS have analyzed the influence of the process parameters’ sintering lamp power, sintering lamp speed, process temperature and amount of ink or RAM applied on the part properties. These investigations have been carried out almost exclusively with the so-called HSS [8,9,10,11,12,13,14,15], which is a modified Sinterstation 2500 CI by DTM, and the prototype/alpha VX200 HSS by voxeljet [16,17,18,19,20]. Only the investigations [21,22] have been carried out using a VX200 HSS by voxeljet. Some of these investigations have focused on materials other than polyamide 12 (PA 12), such as thermoplastic elastomers (TPE) [10,13,15,16], poly(methyl methacrylate) (PMMA) [17] and polypropylene (PP) [18]. Beyond that, Norazman [23] investigate the influence of the type of sintering lamp and Fox [24] investigates the influence of the type of ink on part properties with PA 12.

The following explanations provide an overview of the studies carried out on PA 12 with regard to the influence of the process parameters on the part properties: Majewski et al. [8] analyzed the impact of the sintering lamp and the process temperature by using the HSS. Parts manufactured from PA 12 (Duraform PA by 3D Systems) at a 0° inclination to the build platform were investigated for tensile properties. The overall improvement in tensile properties was demonstrated with increasing process temperature and sintering lamp power, where maximum values for Young’s modulus of ~1400 MPa, ultimate tensile strength of ~34 MPa and elongation at break of ~10% were achieved. Ellis et al. [11] investigated the correlation between the percentage crystallinity φ_c_ (Equation (2)) and the tensile properties of PA 12 tensile bars manufactured at a 0° inclination to the build platform. For this purpose, the specimens were manufactured with different amounts of applied ink. With increasing amounts of applied ink, the percentage crystallinity φ_c_ decreases slightly from ~30% to ~23%. It was observed that a percentage crystallinity φ_c_ of 25% resulted in fully melted particles and maximum stiffness, while lower or higher values of percentage crystallinity φ_c_ resulted in lower stiffness. At a percentage crystallinity φ_c_ of 25%, Young’s modulus is 1550 MPa. In contrast, at a percentage crystallinity φ_c_ of 22%, Young’s modulus is 1200 MPa and at a percentage crystallinity φ_c_ of 28%, Young’s modulus is 600 MPa. A similar trend was observed for tensile strength, where a percentage crystallinity φ_c_ of 23% resulted in an ultimate tensile strength of 45 MPa. Due to the reduced stiffness, the highest elongation at break value was achieved at the lowest obtained percentage crystallinity φ_c_ (21%). Noble et al. [12] also investigated the correlation between the percentage crystallinity φ_c_, part density and tensile properties of PA 12 tensile bars manufactured at a 0° inclination to the build platform. For tensile properties, the same dependence on percentage crystallinity φ_c_ was observed as in [11]. It was observed that a high resp. to high energy triggers degradation of the polymer chains so that, after full melting of the polymer, the percentage crystallinity φ_c_ decreases with an increasing energy input. This would also explain the curves for Young’s modulus and ultimate tensile strength. The highest part density achieved was ~0.98 g/cm^3^. A similar maximum part density (~1.00 g/cm^3^) was determined by Rouholamin et al. for PA 12 (Duraform PA by 3D Systems) parts [14]. Norazman [15] investigated the influence of the amount of ink applied and the type of sintering lamp on the properties of PA 12 (PA2200 by EOS) tensile bars manufactured with the HSS at a 0° inclination to the build platform. The sintering lamp with higher irradiance resulted in maximum values of ~42 MPa for tensile strength and ~1800 MPa for Young’s modulus, as well as a higher part density of about 0.99 g/cm^3^. However, the maximum elongation at break (~12%) was achieved at significantly lower values for ultimate tensile strength and Young’s modulus. Zhu et al. [19,20] investigated the surface roughness, porosity and tensile properties of PA 12 (PA2200 by EOS) tensile bars manufactured with the prototype VX200 HSS at a 0° inclination to the build platform by varying some process parameters. The specimens with the lowest porosity (~5%) achieved the highest ultimate tensile strength (~38 MPa) and elongation at break (~16%). It was also found that surface roughness depends on the orientation of the surface of the specimen in the build chamber. The specimens with the lowest surface roughness have an arithmetical mean height of the surface S_a_ of ~5 µm for the bottom, ~8 µm for the top and ~20 µm for the side of the specimens. Pezold et al. [22] analyzed the influence of the amount of ink on the flexural properties of flexural bars manufactured with the VX200 HSS at a 0° inclination to the build platform. The maximum values obtained were 1500 MPa for the compressive modulus and 60 MPa for the compressive strength.

The influence of part orientation and part position on HSS has hardly been analyzed. Fahad et al. [25,26] analyzed benchmark parts for their geometric accuracy and curling in relation to their position in the build job layout in the z-direction. The parts were made from PA 12 (Duraform PA by 3D Systems) using the HSS. It was observed that the temperature of the build piston had a significant effect on the flatness of the parts. Furthermore, Pezold [21] analyzed the tensile properties of PA 12 (HSS powder PA 12 Type B by voxeljet) tensile bars produced with VX200 HSS at 0°, 45° and 90° inclinations to the build platform using different process parameter settings. It was found that the part orientation tends to have a small effect on the ultimate tensile strength and Young’s modulus and a significant effect on the elongation at break. The maximum values obtained independently are 50 MPa for tensile strength, 1800 MPa for Young’s modulus and 12%, 8% and 7% for elongation at break at 0°, 45° and 90° inclinations. Besides this, Ellis et al. [27] investigated the surface roughness, porosity and tensile properties of PA 11 (DuraForm^®^ EX-Natural by 3D Systems) tensile bars manufactured with the HSS at seven angles of inclination to the build platform (0°, 15°, 30°, 45°, 60°, 75° and 90°). It was observed that, with the exception of surface roughness, all the properties investigated gradually deteriorated as the build angle increased.

### 1.4. Need for Action

Apart from [21,22], no studies have been carried out with the current and commercially available version of the VX200 HSS for PA12, which differs from the prototype resp. alpha VX200 HSS in terms of both software and hardware (e.g., sintering lamp, overhead lamp, ink system, recoating system). Furthermore, with the exception of [21,22], none of the publications used the PA 12 powder certified by voxeljet for the VX200 HSS (HSS powder PA 12 Type B by voxeljet) for the production of parts. A characterization of this PA 12 powder has not yet been carried out. In addition, most studies have used mainly fresh PA 12 powder rather than a mixture of used and virgin powder, as is common in the industry. The properties of PA 12 parts manufactured by HSS that have been frequently analyzed are part density, porosity, percentage crystallinity φ_c_ and tensile properties. There are few publications that have analyzed surface roughness, flexural properties and geometric accuracy. However, none have analyzed the compressive properties, hardness, heat resistance or linear coefficient of thermal expansion. In addition, the influence of the part orientation on the mechanical properties of PA 12 parts has not been widely investigated. Therefore, the aim of this study was to analyze a wide range of properties of PA 12 parts produced on the first commercially available HSS machine, the VX200 HSS, using the PA 12 powder certified for the VX200 HSS in the recommended mix ratio of used to virgin (70 wt% to 30 wt%) and standard process parameters.

## 2. Experimental Methodology

### 2.1. PA 12 Powder and Ink

The PA 12 powder (HSS powder PA 12 Type B by voxeljet) and the ink (HSS ink Type B by voxeljet) used are the standard for the VX200 HSS. The ink consists essentially of a petroleum-based carrier fluid and ~5.6% carbon black, which is used as RAM [21]. Prior to the HSS process, the powder was sieved through a 180 µm mesh and mixed in a PM 10 mixer by Dr. Fritsch Sondermaschinen at a ratio of 70 wt% used powder to 30 wt% virgin powder.

### 2.2. HSS System and Process Parameters

**Figure 2:**
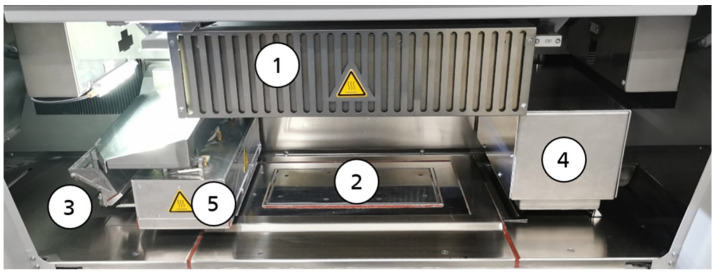
Build chamber of the VX200 HSS (➀ overhead heater, ➁ build chamber, ➂ recoater, ➃ printhead unit, ➄ sintering lamp).

The first commercially available HSS machine, the VX200 HSS, was used for the study. The build chamber of the VX200 HSS, shown in Figure 2, is equipped with a 1.8 kW mid-wavelength infrared ceramic overhead heater ➀ to generate the required process temperature of the powder bed surface during the process. The overhead heater is controlled by comparing the set process temperature with the actual powder bed surface temperature measured by a pyrometer. To avoid thermal process failures such as part curling, the temperature within the powder cake is kept sufficiently high by the heated walls and floor of the build chamber ➁. As with other PBF/P technologies, the applied powder must be pre-heated to reduce the additional thermal effects. Therefore, the heater integrated into the recoater ➂ ensures that the feed powder temperature is reached before the powder is applied to the powder bed surface. The printhead unit ➃, which applies the ink, contains three XAAR 1003 GS6 printheads with 1000 nozzles each. A near-infrared quartz halogen lamp ➄ of 3 kW is used in combination with a filter glass for the selective energy input.

The process parameters (Table 1) were set, as recommended by voxeljet for the PA 12 powder, and kept constant during the HSS process. After the layer-by-layer building process and the cool-down of the build job to room temperature in the build chamber, the specimens were unpacked and glass bead-blasted with a jet pressure of 3 bar.

### 2.3. Specimens and Build Job

The build job is designed to analyze not only the influence of the part orientation but also the part position in the build job. The number of specimens, their orientation, and their position within the build job are listed in Table 2. All samples were at least 10 mm apart.

**Figure 3:**
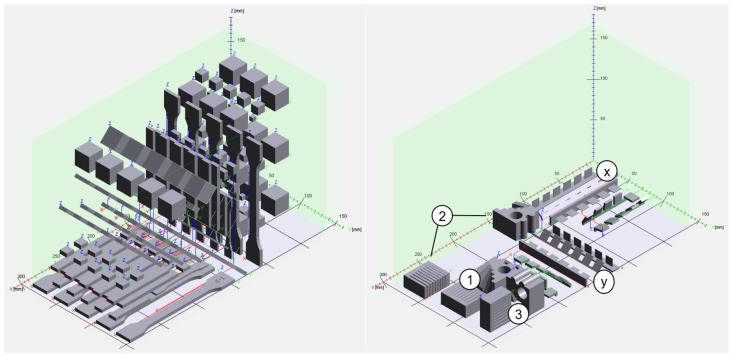
Build job I (**left**) and build job II (**right**).

The specimens listed in Table 2 were manufactured using two processes. Figure 3 shows the build job of these two processes. The specimens and their build orientation are described in more detail below.

## 3. Powder and Sample Analysis Methods

### 3.1. Particle Analysis

The achievable packing density of the powder bed depends, among other things, on the Particle Size Distribution (PSD) of the powder. A more compact powder bed is expected when the PSD contains almost equal proportions of fine and large particles. In the case of PBF/P, large particles lead to better powder flowability, while fine particles lead to a denser powder bed [28]. The particle size analyzer Camsizer XT by Retsch Technology GmbH(Hann, Germany) was used to obtain the volume-based PSD. To compare the virgin powder with the mixed powder (used/virgin of 70 wt%/30 wt%) used in this study, volume-based PSD was determined for both.

The shape and surface characteristics of the powder particles also have a significant effect on the powder bed. If the powder particles are not largely round and the surface is very rough, this can result in inappropriate powder application [28]. The shape and surface were analyzed by using Scanning Electron Microscopy (SEM) images taken on a Quanta 200 by FEI.

### 3.2. Powder Flowability Analysis

Powder flowability is one of the key factors in determining a powder’s suitability for the PBF/P process. It has a significant influence on the recoating process and the surface quality of the powder bed. In addition, it can predetermine the performance of the parts, especially porosity, mechanical properties and surface roughness [28,29]. In this study, the Hausner Ratio H_R_ was determined according to VDI 3405 Part 1.1 [29] to assess the powder flowability. The Hausner ratio H_R_ can be calculated from Equation (1), knowing the bulk density ρ_∞_ and tap density ρ_0_ of the powder. The bulk density ρ_∞_ was measured by using an apparent density tester by Coesfeld. The tap density ρ_0_ was determined using a JEL STAV II tap volumeter by J. Engelsmann, with a 250 mL measuring glass cylinder and a drop height of 3 mm. The powder was tapped 500 times for 2 min. Each density was measured three times for each powder tested.

H_R_ = ρ_0_/ρ_∞_ (1)

### 3.3. Thermal Analysis

In the HSS process, materials are processed under the influence of temperature. Understanding the thermal properties of the material is, therefore, essential. Both powder and specimens were tested to determine their thermal properties and the percentage crystallinity φ_c_ using Differential Scanning Calorimetry (DSC). Samples were prepared by cutting pieces from cubes located at the top, middle and bottom of the build job (Figure 3). Measurements were performed in a calorimeter DSC 1 by Mettler Toledo according to DIN EN ISO 11357-1 [30]. Samples of 10 ± 3 mg were heated from 0 to 220 °C and subsequently cooled back to 0 °C at the same heating and cooling rate of 10 K/min.

The percentage crystallinity φ_c_ of the samples was determined by Equation (2), where ΔH_ms_ is the melting enthalpy of the sample measured by DSC and ΔH_m(100%)_ is the theoretical melting enthalpy of a fully crystalline sample. For the theoretical melting enthalpy for a sample of 100% crystalline PA 12, a melting enthalpy of 209.3 J/g is used [31].

φ_c_ = ΔH_ms_/ΔH_m(100%)_ (2)

In addition, the thermal properties of the specimens were investigated using Thermomechanical Analysis (TMA) to determine the Coefficient of Linear Thermal Expansion (CLTE) α. The CLTE α describes the length change of a material as a function of the temperature. TMA was carried out on the specimens’ cubes small (Table 2) located at the top, middle and bottom regions of the build job (Figure 3). TMA experiments were carried out from room temperature (25 °C) to 160 °C at a heating rate of 1 K/min using a TMA Q400 by TA Instruments. The initial force applied to the sample was 0.05 N. CLTE α was calculated according to Equation (3) [32] for defined temperature ranges ∂T. The change in the sample length ∂l from the initial one l_0_ was measured over these temperature ranges.

α = 1/l_0_∙∂l/∂T (3)

Heat Deflection Temperature (HDT) was tested on bending bars (Table 2), oriented at 0°, 45° and 90° to the xy-plane. Three specimens were tested for each orientation. The tests were performed in accordance with ISO 75-2 [33] using a bending stress of 1.8 MPa on a CEAST HDT VICAT3 by Instron.

### 3.4. Density Analysis

The skeletal density of the PA 12 powder was measured by gas pycnometry. The measurements were conducted under a helium atmosphere in a gas pycnometer AccuPyc II 1340 by Micromeritics Instrument Corporation.

The part density of the specimens was determined according to DIN EN ISO 1183-1 [34] and Archimedes’ principle. Specimens were weighed and then immersed in ethanol. Because PA 12 and water have similar densities of ~1 g/cm^3^, ethanol was used for the density measurement. The density of ethanol was 0.79 g/cm^3^. Cubes large located at the top, middle and bottom regions of the build job layout (Figure 3) were used to measure the part density. The part density of the cubes rotated at 45° to the y-direction was evaluated as well.

### 3.5. Mechanical Analysis

Tensile tests were carried out in accordance with DIN EN ISO 527-1 [35] on a universal testing machine Z020 by ZwickRoell at a test speed of 50 mm/min. In addition to the standard type 1A, smaller tensile bars of the type 1BA were manufactured and analyzed.

Flexural tests were performed using flexural bars (Figure 3) on a testing machine Z2.5 by ZwickRoell applying DIN EN ISO 178 [36].

Compression tests were carried out on a universal testing machine Z050 by ZwickRoell, with a testing speed of 1 mm/min following DIN EN ISO 604, using compression bars (Table 2) [37]. To analyze the influence of the part orientation, tensile, bending, and compression bars were oriented with an inclination of 0, 45, and 90° to the xy-plane, as shown in Figure 3.

Shore D hardness was tested by applying DIN EN ISO 868 [38] using a HD 3000 durometer of type D by Hildebrand on the cubes large (Table 2). To analyze the influence of the part position, specimens on the top, middle and bottom positions in the build job were considered.

### 3.6. Fractured Surface Analysis

The morphology of fractured areas of tensile bars was examined using SEM images taken on an ULTRA PLUS by Carl Zeiss. The inductance of the specimens was increased by sputtering them with gold. Fracture behavior was observed at tensile bars with inclinations of 0°, 45° and 90° to the xy-plane.

### 3.7. Surface Roughness Analysis

For surface roughness analysis, the arithmetical mean height of the surface S_a_ was measured using an optical 3D profilometer VR-5000 by Keyence in accordance with DIN EN 25178 [38]. For the analysis, surface roughness specimens (Figure 3) with varied inclination angles were designed based on VDI 3405 Part 3.2 [39]. The surface roughness specimens covered the range of inclination angles from 0° to 90° with increments of 10°. To evaluate the effect of the direction of powder application, the surface roughness specimens were printed in both x- and y-directions. With the length and the width of 10 mm of each surface inclined, these specimens enabled the arithmetical mean height of the surface S_a_ measurements on the areas of 5 mm × 8 mm. Three measurements were taken of each inclined element on both the top and bottom sides.

### 3.8. Geometric Accuracy Analysis

Geometric accuracy is determined by the deviation of the geometric accuracy specimens from its CAD model. For this purpose, the geometric accuracy specimens are digitized with an ATOS Compact Scan 5M 3D scanner by GOM. The software GOM Inspect Suite 2020 by GOM is then used to perform a surface comparison using the best-fit method. The geometric accuracy specimens are 30 mm × 30 mm × 20 mm and have a hole and many internal and external radii and inclined surfaces to reproduce various of geometries. Three geometric accuracy specimens were manufactured, each with a different orientation in the build job; see Figure 3.

### 3.9. Feasible Geometries Analysis

To analyze the minimum feasible geometric elements, two different specimens (minimum wall thickness specimens and minimum gap dimension specimens) were designed according to the draft of VDI 3405 Part 3.2 [39]. Both were manufactured by different orientations in the build job (Figure 3), regarding the alignment of the specimens in the x- and y-directions and in 0°, 45° and 90° rotations towards the xy-plane. The square wall of the minimum wall thickness specimens had a side length of 10 mm. The different wall thicknesses range from 200 µm to 700 µm in increments of 100 µm. At a 0° rotation, the square wall is oriented parallel to the xy-plane. The minimum gap samples have a wall gap length of 15 mm and a gap height of 25 mm. The gap thicknesses used are 200 µm to 1000 µm in increments of 100 µm.

## 4. Results and Discussion

### 4.1. Characterization of Powder

**Figure 4:**
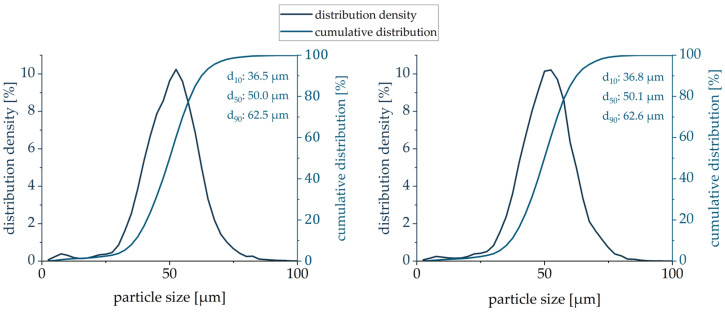
Volume-based PSD for virgin (**left**) and mixed (**right**) PA12 powder.

The obtained volume-based PSD curves of both (virgin, mixed) PA12 powders looked very similar, with a median value d_50_ of 50.0 µm and 50.1 µm, respectively (Figure 4). The volume-based PSD range is sufficiently wide with the presence of both fine and large particles. The volume-based PSD curve is moderately right-shifted, indicating the higher volume fraction of finer particles than that of larger particles. There is a slight decrease in fines when comparing the virgin and mixed powders. As the fine particles contribute to better packing density by filling the space between the larger particles [28], a slightly better compaction of the powder in the powder bed can be expected for the virgin powder.

The relative density of both powders was analyzed to assess the density of the powder volume. The relative density can be described as the ratio of the measured bulk density to the skeletal density of the PA 12 powder. Knowing both densities, the relative density was estimated to be 44% and 42% for virgin and mixed PA 12, respectively. These results correlate with the results of the volume-based PSD. As the fine particles are responsible for better powder compaction [28], their reduction resulted in a lower packing density of the blended powder.

**Figure 5:**
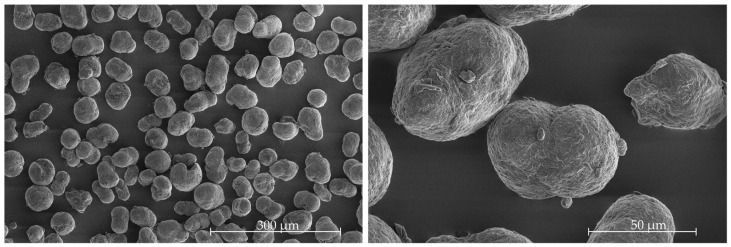
SEM micrographs of the PA 12 powder.

Figure 5 shows SEM images of the powder at different magnifications. The powder shows the typical potato shape, which is a result of the powder being produced by precipitation. Experience has shown that this shape is suitable for achieving good powder flowability in PBF/P processes [28].

The measurements of the Hausner ratio H_R_ resulted in a Hausner ratio H_R_ of 1.14 ± 0.01 for virgin powder and a slightly higher value of 1.16 ± 0.01 for mixed powder. Because the Hausner ratio H_R_ for both powders is less than 1.25, it can be assumed that the powders have good powder flowability [28]. A smooth and homogeneous recoating process can therefore be expected.

### 4.2. Thermal Behavior of Powder and Parts

**Figure 6:**
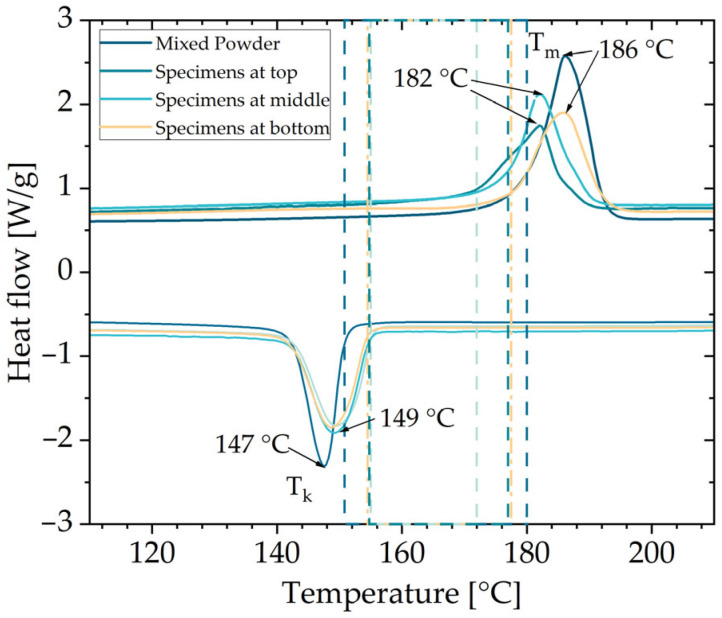
DSC curves of mixed powder and specimens regarding different positions at the build job.

The thermal behavior of the PA 12 samples analyzed by DSC is shown in Figure 6. The range between the onset points of melting (T_m_(onset)) and crystallization (T_K_(onset)) of the powder samples, and therefore the sintering window, is close to 30 °C. This is a typical value for PA 12 powders processed in PBF/P.

The sharp exothermic crystallization peak (T_K_(peak) = 147 °C) of the powder sample shifted to a broader crystallization peak (T_K_(peak) = 149 °C) of the specimens’ samples. Such a peak shift can be caused by nucleating agents such as degraded polymer chains or carbon black. In the case of HSS, it can be an indicator of carbon black, remaining in the specimens [40,41]. All melting peaks (T_m_(peak)) showed a single peak behavior, indicating a complete melting of the PA 12 powder particles [42,43,44]. However, the endothermic melting peak shifted from 186 °C for the powder sample to flatter and broader peaks of 182 °C for the specimens’ samples. According to Rosso et al. [45], the shift towards lower melting temperatures can be explained by the formation of the α-phase of PA 12, which has a lower melting temperature of 175 °C. There was no apparent correlation between the position of the specimens in the build job and the peak shift.

The results for melting enthalpy H_ms_ and percentage crystallinity φ_c_ are shown in Table 3. The specimens had a lower melting enthalpy than the powder. Riedelbauch et al. [41] found a similar decrease in melting enthalpy for specimens produced by MJF. The percentage crystallinity φ_c_ of the specimens decreased significantly from 48.5% to ~33.5% compared to the powder. The same behavior was observed in different PBF/P processes [41,42,43,45,46]. A slight increase in percentage crystallinity φ_c_ with a closer position to the building plate was observed. Apparently, the duration of the thermal impact within the process influences the thermal post-crystallisation, which explains the higher percentage crystallinity φ_c_ values for the specimens that were produced first and remained in the powder cake longer, thus having lower cooling rates [47,48]. It should be noted that the bottom temperature of the build chamber is 175 °C throughout the whole layer-by-layer building process. However, the percentage crystallinity φ_c_ is higher than that previously measured for PA 12 parts manufactured with the HSS [11,12].

**Figure 7:**
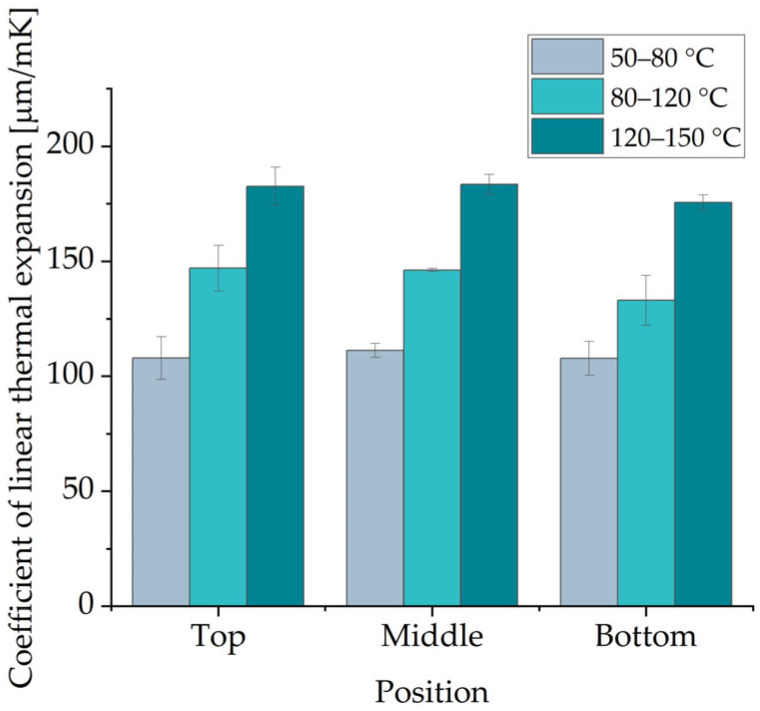
CLTE α of specimens regarding their position in the build job.

The change in length of the specimens as a function of the temperature showed linear behavior in the following temperature ranges: 50–80 °C, 80–120 °C and 120–150 °C. The CLTE α values for these temperature ranges are shown in Figure 7. While the position of the specimens in the build chamber has no significant effect on the CLTE α, a clear difference between the temperature ranges and the CLTE α was observed. Higher CLTE α values, up to ~75%, were found in the temperature range of 120–150 °C compared to those in the range of 50–80 °C.

**Figure 8:**
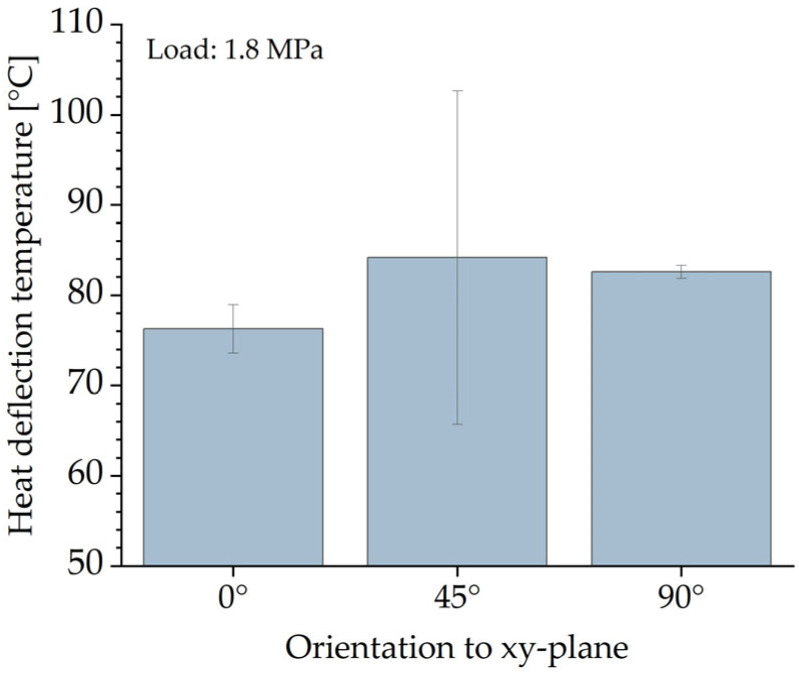
HDT of specimens regarding their orientation in the build job.

The HDT shows the highest temperature values at which the specimens can withstand the given load. As shown in Figure 8, the orientation of the specimens had a clear influence on the HDT at 1.8 MPa. It was rather unexpected that the specimens manufactured without any inclination had the lowest HDT of ~76 °C, about 10 °C lower than the HDT value obtained for the specimens fabricated with a 90° inclination. The maximum HDT value was obtained from the 45° inclined specimens at ~84 °C. Due to the highest standard deviation of almost ±20 °C, the results were examined carefully without drawing any conclusions. Such a high deviation could be explained by the low reproducibility of the specimen properties. Regardless of the orientation of the specimens, PA 12 specimens made with HSS can withstand at least ~75 °C when loaded with 1.8 MPa.

### 4.3. Density and Porosity of Parts

The skeletal density of the mixed powder analyzed by gas pycnometry is 1.068 g/cm^3^. The part density analysed using the Archimedes principle is ~1.03 g/cm^3^. No significant difference in part density was found between the position and orientation of the specimens in the build job. It can therefore be assumed that the melt formation was almost identical throughout the whole layer-by-layer build process. By comparing the skeletal density of the powder with the part density, the porosity of the specimens is ~3.6%. The part density measured is similar to or slightly higher than that previously achieved in HSS for PA 12 with the HSS [12,14,15] and the prototype VX200 HSS [19,20]. Furthermore, according to some studies, the values achieved are comparable to those achieved with LS [46,49,50] and MJF [51].

### 4.4. Mechanical Properties of Parts

**Figure 9:**
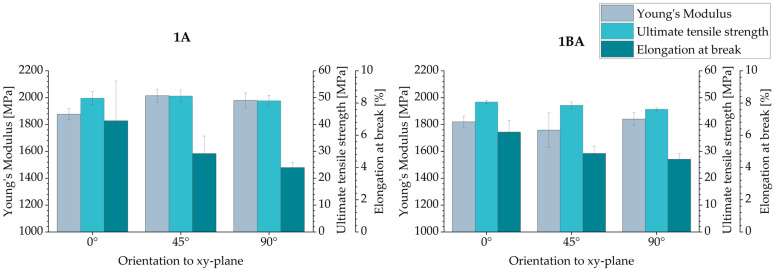
Tensile properties of type 1A and 1BA specimens regarding their orientation in the build job.

The tensile properties of tensile bars 1A and 1BA were considered as a function of their orientation in the xy-plane. The values obtained for Young’s modulus, ultimate tensile strength and elongation at break are shown in Figure 9. The tensile test results and selected literature results for HSS, MJF and LS are also given in Table 4.

When comparing the results of the two types of tensile bars, it was observed that the smaller tensile pars (1BA) tended to have slightly lower values of Young’s modulus and ultimate tensile strength. The most significant difference in Young’s modulus between 1A and 1BA tensile bars was observed for tensile bars with an inclination of 45°. An approximately 15% higher Young’s modulus was obtained for type 1A than for 1BA. Due to the smaller size, 1BA tensile bars have a smaller-cross section and a larger circumference-to-area ratio than 1A tensile bars. This could lead to an increased effect of possible cross-sectional defects and an increased surface notch effect, which could negatively affect the mechanical properties of the 1BA tensile bars compared to the 1A tensile bars. The impact of the notch effect could be particularly significant for specimens with a 45° inclination. The difference in elongation at break was less obvious. Both types of tensile bars showed almost similar results, which were within the standard deviation.

No significant difference was observed in the ultimate tensile strength of the 1A tensile bars with respect to their orientation. Only a slight decrease in the ultimate tensile strength values of the 1BA tensile bars was observed as the angle of inclination to the xy-plane increased. A slight decrease in the ultimate tensile strength is also shown in the Material Data Sheet (MDS) (Table 4) and achieved by [21]. For the 1BA tensile bar, no significant difference in Young’s modulus was observed. For the 1A tensile bars, those manufactured with an inclination of 0° have a slightly lower value than those with an inclination of 45° and 90°. This also correlates with the values from the data sheet and the analysis of [21]. The elongation at break decreases significantly for both types of tensile bars with an increasing angle of inclination of the tensile bars to the xy-plane. This behavior is also consistent with the analyses of [21] and the values for 0° and 90° inclined specimens given in the MDS (Table 4).

A similar effect of part orientation on the tensile properties can be observed for specimens manufactured via MJF (Table 4). While a reduction in part properties with an increasing angle of inclination is more typical for other AM technologies including LS, MJF parts generally showed an improvement in tensile strength and Young’s modulus for specimens inclined at 90° compared to 0° [40,42,51,52].

When comparing the results with those achieved using the HSS and the prototype VX200 HSS for PA 12, the Young’s modulus and tensile strength achieved in this work are in most cases significantly higher. However, the elongation at break is significantly lower. Compared to the MDS (Table 4) and the analyses of [21], the values for Young’s modulus are slightly higher, those for tensile strength are similar and those for elongation at break are significantly lower. This behavior may be due to the relatively high-percentage crystallinity φ_c_ of the test specimens in this work, since the elongation at break decreases with increasing percentage crystallinity φ_c_, as described in [11,12].

Comparing the results with those achieved with MJF or LS, summarized in Table 4, this study obtained basically similar ultimate tensile strength values for HSS. The Young’s modulus obtained in this study was ~25% higher than the MJF and comparable to the LS. A significant difference in elongation at break was observed between these three PBF/P processes. However, the values obtained for elongation at break were significantly lower than those obtained for MJF or LS.

**Table 4:** Comparison of selected tensile properties achieved with PA12 in LS, MJF and HSS.

	Young’s Modulus [MPa]		Ultimate Tensile Strength [MPa]		Elongation at Break [%]	
	xy	z	xy	z	xy	z
LS [53]	1674 ± 15	1713 ± 14	45.58 ± 0.36	46.92 ± 0.20	13.07 ± 1.88	14.41 ± 1.46
LS [54]	2070 ± 60	2035 ± 32	53.22 ± 1.14	50.70 ± 0.71	9.66 ± 3.47	8.70 ± 1.84
MJF [53]	1466 ± 20	1810 ± 25	42.95 ± 1.09	51.59 ± 0.60	15.80 ± 1.39	17.77 ± 0.49
MJF [55]	1128 ± 68	1337 ± 98	45.80 ± 3.50	47.90 ± 0.90	11.20 ± 1.80	11.40 ± 1.30
MJF [51]	1242 ± 28	1246 ± 37	47.00 ± 0.90	49.00 ± 0.60	19.00 ± 2.80	16.00 ± 1.90
HSS [8]	1400	-	34	-	10	-
HSS [19]	1793	-	44.40	-	13.50	-
HSS [23]	1720	-	42.20	-	11.70	-
HSS [24]	1127 & 1615	-	37.30 & 42.70	-	20.80 & 18.60	-
HSS MDS [56]	1716 ± 89	1725 ± 59	52.00 ± 1.00	46.00 ± 2.00	10.00 ± 1.00	5.00 ± 1.00
HSS current work Type 1A	1876 ± 40	1979 ± 56	49.81 ± 2.45	48.86 ± 1.99	6.90 ± 2.47	3.99 ± 0.32
HSS current work Type 1BA	1820 ± 41	1840 ± 50	48.39 ± 0.51	45.67 ± 0.50	6.08 ± 0.61	4.47 ± 0.35

**Figure 10:**
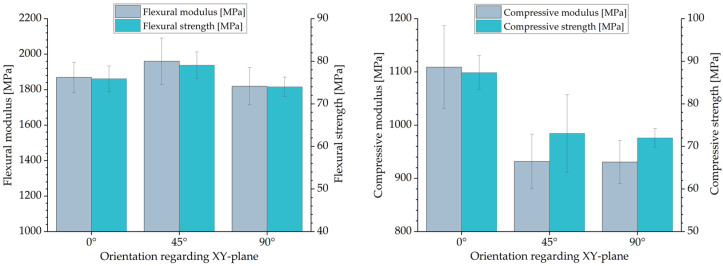
Flexural-(**left**) and compressive (**right**) properties regarding their orientation in the build job.

The results of the flexural and compression tests are shown in Figure 10. The bending pars with an inclination of 45° showed the highest values of ~1960 MPa and ~80 MPa for flexural modulus and flexural strength, respectively. The flexural properties obtained for the 0° and 90° specimens were within the standard deviation with a flexural modulus and flexural strength of ~1850 MPa and ~75 MPa, respectively. Compared to MJF [51], the achieved results for the flexural modulus and flexural strength are higher.

The compression tests showed similar values of the compressive modulus and compressive strength for specimens with an inclination of 45° and 90°. A significant increase in the compressive modulus and compressive strength was observed for specimens with an inclination of 0° compared to 45° and 90°. In this case, the compressive modulus and compressive strength increased by ~18% from ~930 MPa to ~1100 MPa and by ~21% from ~72 MPa to ~87 MPa, respectively. For HSS, the compressive properties may be dependent on the size of the cross-sectional area, with higher compressive values being obtained for larger areas. Such a dependence of the compressive behavior on the size of the specimens has been observed for LS by Tomanik et al. [57].

To analyze the hardness, Shore D Hardness was determined. Considering the standard deviation, the results do not show any significant differences regarding the position of the specimens in the build job layout. The average Shore D Hardness determined is ~73.

### 4.5. Fractured Surface of Parts

**Figure 11:**
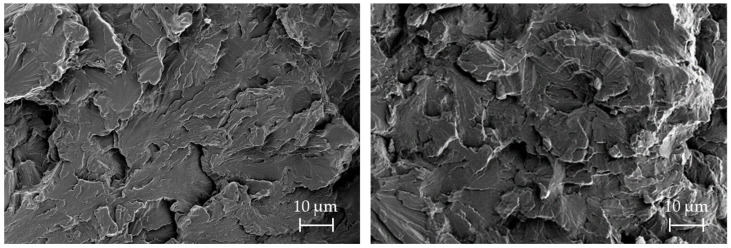
SEM micrographs of the fractured surface of the tensile specimens orientated with 90° (**left**) and 45° (**right**) to the x-y-plane.

**Figure 12:**
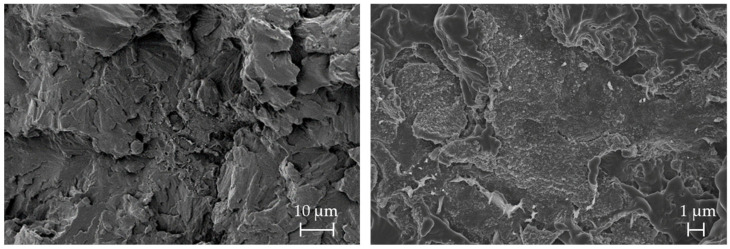
SEM micrographs of the fractured surface of the tensile specimens orientated with 0° (**left**) to the x-y-plane and an enlarged view of the carbon black (**right**).

After the tensile tests, the fracture surface of the tensile bars was examined by SEM. SEM images of the tensile bars confirmed the low elongation at break, compared in Table 4, by showing brittle fracture behavior. The stepped brittle fracture bands [58] were particularly well observed in the 90° specimens (Figure 11, left) and appear to propagate within a plane fracture surface. For the 45° specimens (Figure 11, right), the fracture surface showed a more uneven but also brittle behavior, spreading more radially and showing similarities to a shear fracture. In the SEM micrograph of the 0° specimen (Figure 12, left), in addition to the brittle fracture paths, areas with deviating fracture surfaces could be detected. The magnified image (Figure 12, right) shows structures approximately 50 nm in diameter, which are thought to be carbon black of the ink. At the interface, the PA 12 fracture surface appears to be smeared rather than sharp-edged, suggesting an increase in the ductility of the specimen.

### 4.6. Surface Roughness of Parts

**Figure 13:**
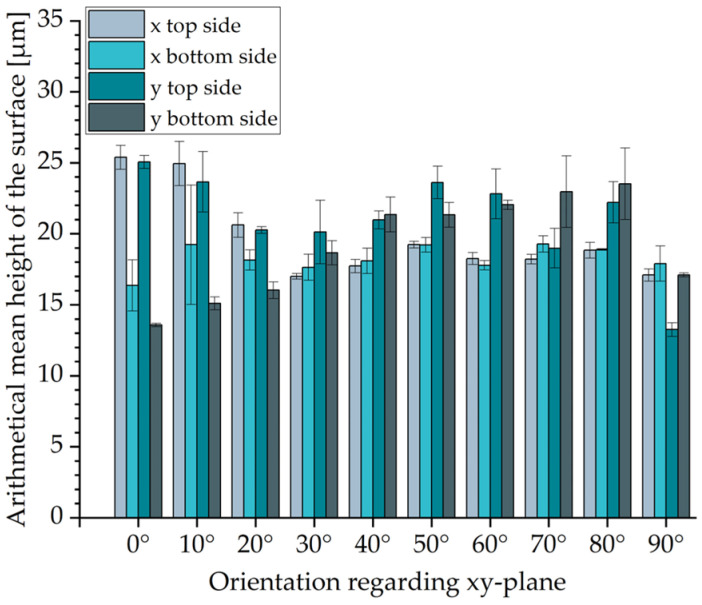
Arithmetical mean height of the surface S_a_ regarding its orientation in the build job.

For the surface roughness, the arithmetical mean height of the surface S_a_ of the top and bottom sides of all segments of the surface roughness specimens was analyzed after the glass bead blasting of the specimens. The values obtained are shown in Figure 13. A distinction is made between the orientations of the specimens in the xy-plane. The specimens were oriented either in the x-direction, in which the recoater applied the powder, or in the y-direction, transverse to the movement of the recoater.

For the inclination of 30° to 80° regarding the xy plane, a difference between the orientations of the specimens in the x- and y-directions can be seen. Parts oriented in the x-direction achieve lower values than those oriented in the y-orientation. This is probably due to the influence of the direction of the movement of the recoater. Furthermore, there is no significant difference between the top and bottom sides of the segments with an inclination of 30° to 80°. For inclinations from 0° to 20°, the top side of the segment has a significantly higher value. At a 90° inclination and orientation in the x-direction, there is no significant difference between the top and bottom sides. This is because both are perpendicular to the xy-plane and parallel to the movement of the recoater. Because this is not the case in the y-direction, there is a significant difference between the top and bottom sides. The highest value (~25 µm) was measured on the top side of the segments positioned horizontally (0°) to the xy plane. The lowest value (~14 µm) was measured on the bottom side of the segment positioned horizontally (0°) and on the top side of the segment positioned vertically (90°) in the xy-plane.

Guo showed that parts manufactured by MJF have a smoother bottom than the top surface. Measurements of the arithmetical mean height of the surface Sa gave values of 15.2 µm for the top surface and 11.2 µm for the bottom surface [59]. According to VDI 3405 Part 3.2 [39], the highest surface roughness of LS parts can be observed at an angle of inclination of 0° and the lowest can be observed at 90°. When categorizing the results, it must be taken into account that the powder removal process has a major influence on the surface roughness of the components.

### 4.7. Geometric Accuracy of Parts

**Figure 14:**
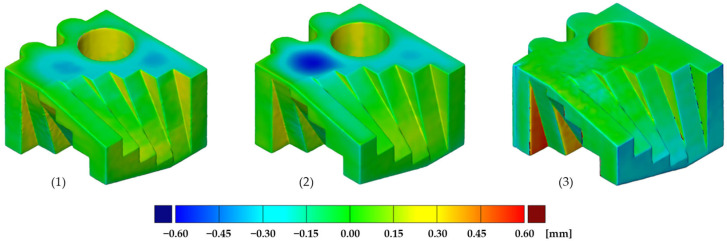
Geometric deviation of the geometric accuracy specimens oriented in the x direction (1) (**left**), y direction (2) (**middle**) and z direction (3) (**right**), displayed as a false-color image.

The results of the geometric accuracy analysis are shown as a false color image of the geometric accuracy specimens oriented in the x- (1), y- (2) and z-orientations (3) within the build job (Figure 14). Specimens (1) and (2), where the axis of rotation of the hole is in the z-direction, show a maximum dimensional increase of +0.30 mm. The maximum dimensional reduction of −0.60 mm on the top of the specimens is assumed to be due to higher shrinkage due to intense energy input in the bulk areas. Specimen (3), where the axis of rotation of the hole is oriented in the x-direction, shows a high accuracy on the top and side surfaces. On the other hand, the surfaces oriented parallel to the xy-plane, and therefore manufactured in the z-direction, show deviations of +0.60 mm and −0.30 mm. The accuracy in the z-direction is reduced by adapting the geometry of the sample for manufacturing in relation to the layer thickness.

### 4.8. Feasible Geometries of Parts

The minimum wall thickness of the specimens was examined in relation to their position within and rotation to the xy-plane (Table 5). Except for a feasibility of 0.4 mm, there is no difference between the placement of the specimens within the xy-plane. In contrast, there is a large difference between the different rotations. While a wall thickness of 0.2 mm is feasible for 0° rotations, only 0.3 mm is feasible for 45° rotations and only 0.5 mm is feasible for 90° rotations. The slicing software, which is used to slice the layout into 80 µm thick layers, stores the information in the coloring of the pixels in bitmaps. Depending on the geometry of the sample, the slicing software uses the colour of each pixel to decide whether to print ink on top of the powder bed or not. In our case, the minimum wall thickness is limited by the combination of the slicing software and the resolution of the print heads.

The minimum gap dimensions for specimens (1) and (2) oriented in the xy-plane showed that wall gaps of 1000 µm were feasible with respect to the described unpacking and glass bead blasting process. On the other hand, for specimen (3), oriented so that the gaps were parallel to the xy-plane, gaps of 700 µm were feasible. It should be noted that, in addition to glass bead blasting, more mechanical removal of the powder between the gaps would result in smaller feasible gaps.

## 5. Conclusions

The present study provides the first comprehensive overview of the properties of PA 12 parts produced on the first commercially available HSS machine, the VX200 HSS. This includes the analysis of the influence of part orientation and, in some cases, part position in the build job layout. Parts were produced using standard PA 12 powder for the VX200 HSS, with a used to virgin powder ratio of 70 wt% to 30 wt% and standard process parameters.

The tensile performance of the parts was analyzed on two kinds of tensile bars. The smaller tensile bars (1BA) had lower Young’s modulus and ultimate tensile strength values. The larger tensile bars (1A) with an inclination of 0° showed the highest ultimate tensile strength (~50 MPa). Basically, no significant difference in ultimate tensile strength was observed with regard to part orientation (0°, 45°, 90°). For Young’s modulus, the difference was also marginal and less than 5%. The highest Young’s modulus (~1979 MPa) was obtained for tensile bars 1A manufactured with an inclination of 45°. On the other hand, part orientation has a significant influence on the elongation at break. For tensile bar 1A, the elongation at break decreases from ~6.90% at a 0° inclination to 3.99% at a 90° inclination. The bending tests showed almost isotropic behavior. However, bending bars with an inclination of 45° had slightly higher values of ~1960 MPa and ~80 MPa for flexural modulus and flexural strength, respectively. The values of the compressive modulus and compressive strength for specimens with an inclination of 45° and 90° are similar. In contrast, specimens with an inclination of 0° show significantly higher values, with a compressive modulus of ~1100 MPa and compressive modulus of ~87 MPa. No influence of the part position in the build job was mentioned for Shore D Hardness, which remained almost constant at ~73. This is also the case for part density and CLTE α. The determined part density is ~1.03 g/cm^3^, which corresponds to a part porosity of 3.5%. CLTE α demonstrated an expansion of the samples with increased temperatures from 100·10^−6^ 1/K at 50–80 °C to 175·10^−6^ 1/K at 120–150 °C. In contrast, the HDT is dependent on the part orientation. The lowest HDT at 1.8 MPa was ~76 °C for specimens with an inclination of 0°; specimens with an inclination of 90° showed values about 10 °C higher. In terms of surface roughness, the results show that the orientation of the part to the xy-plane and, partially, the orientation of the part to the movement of the recoater have an effect on surface roughness. The determined arithmetical mean height of the surface S_a_ is between 14 µm and 25 µm. In terms of geometric accuracy, it was found that this was dependent on the orientation of the parts. This is also the case for the feasible geometries of the parts.

In summary, the properties of the parts are largely similar to those produced by other PBF/P processes. However, there are also significant differences for a few properties. It was also determined that the anisotropy of the parts produced by the VX200 HSS is very low compared to many other additive manufacturing processes.

## Figures and Tables

**Table 1:** HSS process parameters used in the study.

Parameter	Unit	Value
process temperature	°C	173
floor temperature	°C	175
wall temperature	°C	140
recoater speed	m/s	0.1
recoater temperature	°C	130
recoater gap	mm	2.8
recoater intensity of vibration	%	85
sintering lamp speed	m/s	0.15
sintering lamp power	%	100
printhead speed	m/s	0.39
applied ink	pl/mm^2^	3618
layer thickness	mm	0.08
empty layers start	-	100
empty layers cover	-	40
cool-down end temperature	°C	90

**Table 2:** Specimens used in the study.

Specimen	Number (Total)	Position/Orientation	Build Job
cubes large (20 × 20 × 20 mm)	6 × each (18) 5 × each (10)	bottom, middle, top 0°, 45°	I
cubes small (5 × 5 × 5 mm)	5 × each (15)	bottom, middle, top	I
tensile bars type 1A	5 × each (15)	0°, 45°, 90°	I
tensile bars type 1BA]	5 × each (15)	0°, 45°, 90°	I
bending bars (80 × 10 × 4 mm)	10 × each (30)	0°, 45°, 90°	I
compression bars type B	5 × each (15)	0°, 45°, 90°	I
surface roughness specimens	2 × each (4)	x- and y-orientation	II
geometric accuracy specimens	1 × each (3)	orientation ➀, ➁, ➂	II
minimum wall thickness specimens	1 × each (6)	0°, 45°, 90° for x- and y-orientation	II
minimum gap dimension specimens	1 × each (3)	orientation ➀, ➁, ➂	II

**Table 3:** Melting enthalpy and percentage crystallinity φ_c_ of PA12 mixed powder and specimens.

Sample	Melting Enthalpy H_ms_ [J/g]	Percentage Crystallinity φ_c_ [%]
mixed powder	101.9	48.7
specimens at top	69.3	33.1
specimens at middle	70.9	33.9
specimens at bottom	71.6	34.2

**Table 5:** Feasibility of minimum wall thickness regarding the orientation of the parts.

Orientation												
Wall thickness [mm]	0.2	0.3	0.4	0.5	0.6	0.7	0.2	0.3	0.4	0.5	0.6	0.7
90°				■	■	■			■	■	■	■
45°		■	■	■	■	■		■	■	■	■	■
0°	■	■	■	■	■	■	■	■	■	■	■	■

■ feasible wall thickness.

## Data Availability

The original contributions presented in this study are included in the article material. Further inquiries can be directed to the corresponding author.
